# Conservation of Cell Communication Systems in Invertebrate Host–Defence Mechanisms: Possible Role in Immunity and Disease

**DOI:** 10.3390/biology9080234

**Published:** 2020-08-18

**Authors:** Manon Auguste, Teresa Balbi, Caterina Ciacci, Laura Canesi

**Affiliations:** 1 Department of Earth Environment and Life Sciences (DISTAV), University of Genoa, 16136 Genoa, Italy; manon.auguste@edu.unige.it (M.A.); teresa.balbi@unige.it (T.B.); 2 Department of Biomolecular Sciences (DIBS), University “Carlo Bo” of Urbino, 61029 Urbino, Italy; caterina.ciacci@uniurb.it

**Keywords:** innate immunity, invertebrates, extracellular traps, exosomes, tunnelling nanotubes, cell communication

## Abstract

Innate immunity is continuously revealing multiple and highly conserved host–defence mechanisms. Studies on mammalian immunocytes are showing different communication systems that may play a role in coordinating innate immune responses also in invertebrates. Extracellular traps (ETs) are an immune response by which cells release net-like material, including DNA, histones and proteins. ETs are thought to immobilise and kill microorganisms, but are also involved in inflammation and autoimmune disease. Immune cells are also known to communicate through extracellular vesicles secreted in the extracellular environment or exosomes, which can carry a variety of different signalling molecules. Tunnelling nanotubes (TNTs) represent a direct cell-to-cell communication over a long distance, that allow for bi- or uni-directional transfer of cellular components between cells. Their functional role in a number of physio-pathological processes, including immune responses and pathogen transfer, has been underlined. Although ETs, exosomes, and TNTs have been described in invertebrate species, their possible role in immune responses is not fully understood. In this work, available data on these communication systems are summarised, in an attempt to provide basic information for further studies on their relevance in invertebrate immunity and disease.

## 1. Introduction

**Figure 1:**
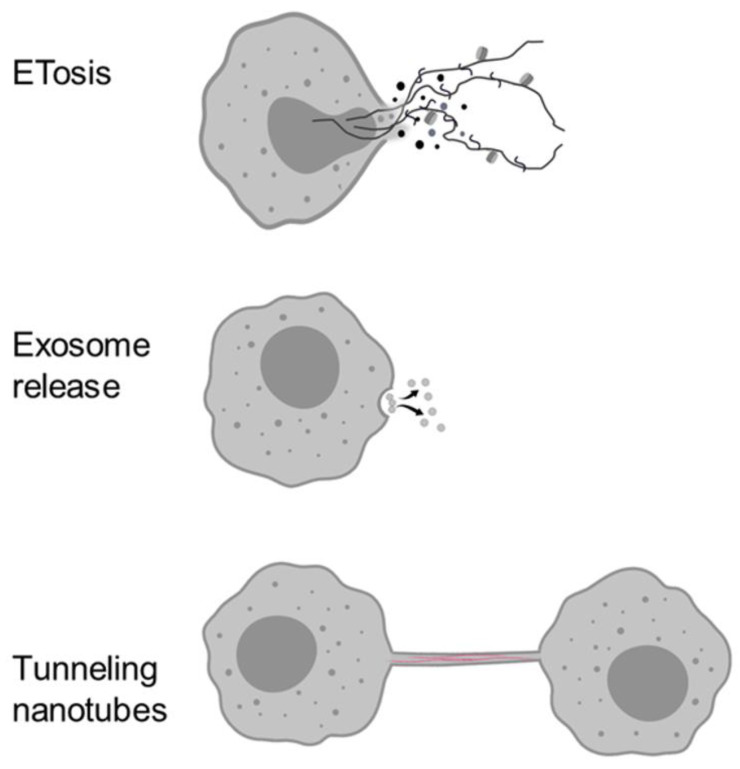
Different cell structures that allow cells to communicate and perform diverse roles in host defence and homeostasis. Top: Extracellular Traps (ETosis), Middle: Exosome release, Bottom: Tunnelling nanotubes (TNTs).

In multicellular organisms, the endocytic and secretory pathways evolved to control all aspects of cell physiology and intercellular communication (immune response, development, hormone-mediated signal transduction, neurotransmission). In mammalian systems, the complexity of the molecular interactions underlying these pathways suggests that a great evolutionary effort has been spent on regulating the cellular response to a variety of different endogenous and environmental stimuli, including immune responses to microbial infection. In particular, innate immunity is continuously revealing multiple and highly conserved host–defence mechanisms. Studies on mammalian immunocytes show different communication systems that may play a role in coordinating innate immune responses also in invertebrates. In this work, the main intercellular communication systems that can play a role in innate immunity, i.e., extracellular traps, exosomes, tunnelling nanotubes, are briefly described (Figure 1), and available knowledge on their role in the invertebrate host response to infection is summarised.

## 2. Extracellular Traps (Etosis)

Extracellular traps (ETs), first discovered in neutrophils in 2004 [1], and therefore, termed NETs, are web-like structures composed of decondensed chromatin heavily impregnated with different antimicrobial proteins that capture, neutralise and kill a variety of pathogens. The main role of ETs is the immobilisation of microbes, which prevents dissemination, and their exposure to a high, localised concentration of antimicrobial proteins.

The generation and release of ETs (NETs, in particular) has been shown to be induced by a variety of internal and/or pathogen-derived molecular signals, including pro-inflammatory cytokines, lipopolysaccharides (LPS), formylated peptides and pharmacological agents [2]. These can lead to stimulation of multiple signalling pathways, that converge on the production of reactive oxygen species (ROS) and nitric oxide [2] that, in turn, induce nuclear/mitochondrial/membrane rupture, followed by proteolytic cleavage, histone deamination, chromatin decondensation and eventual release. Although production of ROS apparently represents an integral part of most of the reaction cascades entailing the release of ETs, NET formation is induced by both NADPH oxidase (NOX) dependent and independent (Ca^2+^ mediated) pathways, depending on the type of stimulus (reviewed in Reference [3]). Autophagy, the original mechanism through which eukaryotic cells acted to resist the invasion of pathogens, has been shown to play a key role in NETs: Autophagic processes not only actively participate in their formation, but also in inhibiting the excessive release of ETs (reviewed in References [2,4].

NET formation is widely regarded as an important part of the mammalian inflammatory repertoire; moreover, NETs have been implicated in a number of pathological conditions, such as fibrosis, thrombosis, autoinflammatory diseases, and cancer progression, through specific protein expression and post-translational modifications (reviewed in Reference [5]). The identification of stimuli that modulate the release of NETs and associated molecules in different physiological and pathological conditions can reveal their role in immune protection, inflammatory and autoimmune diseases and cancer [6].

ETs are produced not only by neutrophils, but also by other immune cell types, such as monocytes and macrophages, eosinophils, basophils, and mast cells [7,8]. In addition to the presence of DNA, they contain associated molecules that can be specific depending on the cell type and on the stimuli [9]. Although ETs play a beneficial role in host defences, they consist of a filamentous network of chromosomal and/or mitochondrial DNA released from the cell after the breakdown of the nuclear/mitochondrial membrane, usually leading to suicidal death, distinct from apoptosis or necrosis (reviewed in References [2,10]). Moreover, the presence of DNA and various enzymes can make ETs harmful, especially if they persist for a long period of time (reviewed in Reference [2]).

However, at least in vitro, ETs have been shown to influence the behaviour of immune cells: NETs are able to down regulate LPS-induced activation of monocyte derived dendritic cells, or can induce macrophage and dendritic cells death, which may limit the ongoing inflammation. Although it is still unknown which components of NETs are responsible for these effects, the fact that persisting ETs can modify molecular and cellular components of the immune system indicates that fast clearing of ETs is extremely important for the proper functioning of the immune response [11]. Recent evidence indicates that ETs tend to aggregate and form larger functional units endowed with a plethora of enzymatic activities that can modify biomolecules at the site of inflammation. ETs, thus, participate in both the initiation and in the resolution of inflammation. Those that escape clearance in the body might challenge immune tolerance and serve as autoantigen repositories that trigger the onset and promote the chronicity of autoimmune diseases [8].

In a broader light, ETs can represent a noncanonical intercellular communication in immune response [7]. During the last decade, evidence has accumulated showing that ETs play a crucial role in the defence mechanisms of various cell types, not only in mammals, but also in other vertebrates, as well as in invertebrates and plants, suggesting that ETs are one of the primordial and evolutionary ancient mechanism of host defence [12]. Many studies are available in marine invertebrates, where ETs have been first described in crustacean haemocytes. ET formation was observed in haemocytes of the Pacific White shrimp *Litopenaeus vannamei* after stimulation with bacterial lipopolysaccharide (LPS), phorbol 12-myristate 13-acetate (PMA) and *E. coli* [13,14]. These studies demonstrated that released of DNA fibres are important for ET-mediated bacterial clearance. Moreover, it was shown that ETs represented a more effective antibacterial response than phagocytosis at high bacterial densities [14]. In the haemocytes of kuruma shrimp (*Marsupenaeus japonicus*) ET formation was also stimulated by peptidoglycan (PGN), and showed that released c-type lysozyme was another component of the antimicrobial response [15].

A comparative study on ET formation by different stimuli was carried out in crab, mussel and echinoderm haemocytes [16]. The results demonstrated the release of chromatin, antibacterial histones and other haemocyte-derived defence factors. In the Pacific oyster *Crassostrea gigas*, haemocytes form ETs associated with antimicrobial histones both in vitro and in vivo, in response to infections and tissue damage, and can further entrap bacteria. These data indicated that, in marine bivalves, ETs participate in host defence by capturing large numbers of microbes and preventing their dissemination [17]. Similar to vertebrate neutrophils, the formation of ETs by oyster haemocytes was dependent on the production of ROS; however, unlike in other species, PMA failed to trigger the oxidative burst and the formation of ETs [18]. In the colonial ascidian *Botryllus schlosseri*, haemocytes have been shown to release amyloidogenic ETs to prevent the spreading of microbes in the case of infection [19]. In a recent study, Romero et al. (2020) [20] first described a robust and reproducible model for the induction, analysis and quantification of ETs production by the haemocytes of the mussel *Mytilus galloprovincialis*. It was demonstrated that in mussel haemocytes, as in neutrophils, the formation of ETs can be triggered through NOX-dependent and NOX-independent pathways, depending on the stimuli. Haemocyte treatment with UV light, the calcium ionophore A23187 and zymosan induced the release of ETs, whereas exposure to *E. coli* and LPS elicited a highly variable response, and PMA and poly I:C were ineffective. Moreover, the results indicate that the release of ET in bivalves may probably depend not only on the type of stimulus, but also on the dose, timing and species analysed, as well as the immunological status of the animal. This variability in the response suggests the presence of the two main forms of Etosis (vital and suicidal) in mussel haemocytes as in mammalian neutrophils [2,10,21]. However, the results also indicate that in bivalves the triggering characteristics and timing may be distinct from those of vertebrate immunocytes. Although in vitro data may not reflect the full capacity of ET formation by bivalve haemocytes, they may help our understanding regarding which haemocyte subtypes are involved in the process. ETs containing DNA and antimicrobial histones were identified in oyster after in vivo bacterial infection and tissue damage [17]. ET formation was highly dependent on ROS production, not only by haemocytes, but probably also on both ROS and damage-associated molecular patterns (DAMPs) or other signals released by inflamed and injured tissues. In this light, both experimental approaches are needed to elucidate the mechanisms of ET formation.

In vivo experiments in crabs (*Carcinus maenas*) also showed that ETs represent a scaffold for assembling haemocytes during encapsulation in gills, a response that sequesters and kills potential pathogens infecting the body cavity and tissues, with different haemocyte subtypes playing different roles in the process [16]. Finally, it should be considered that invertebrate haemocytes are involved not only in immune response, but also in other functions, including the coagulation of haemolymph [22], blurring the line between immunity and haemostasis. In analogy with invertebrate haemocytes, mammalian platelets have increasingly been appreciated as immune cells in recent years. Platelets have been shown to induce NET formation; and in turn, NET components further regulate platelet and neutrophil function. This complex interplay seems to ultimately underlie NET-induced immunopathology (reviewed in Reference [23]). Although the haemocyte sub-populations involved in haemolymph clotting have not been identified yet, in vivo studies may help our understanding the involvement of ETs in communication among different haemocyte sub-populations to perform multiple, integrated functions.

Available knowledge in humans indicates that the fine balance between protective ET formation, and subsequent efficient elimination, implying intercellular communication among different immune cell types, defines the protective versus the detrimental consequences of ET formation in different physio-pathological conditions. Therefore, comparing data on ETs from different phylogenetic groups will contribute to elucidate their conserved role in immunity, health and disease [12].

## 3. Exosomes

Exosomes are defined as secreted membrane vesicles between 30 and 150 nm in size, generated through an endosomal pathway where multivesicular bodies, formed by the maturation of early endosomes into late endosomes, fuse with the plasma membrane (reviewed in Reference [24]). Exosomes are characterised by the presence of different markers, including transmembrane, lipid-bound extracellular proteins (CD9, CD63, CD81, integrins), cytosolic proteins (endosome, membrane-binding proteins, synthenins), and other proteins (calnexin, histones, cytochrome c) [24]. Exosomes are key players in cellular communication, carrying source-specific molecules, such as proteins, growth factors, miRNA/mRNA, among others; their cargo can depend on the cell type, phenotype, and metabolic status. Over the last decades, exosomes have been described to play emerging roles in a number of physiological or regenerative processes, infection and disease; therefore, they have been largely investigated in biomedical research, due to their potential applications in cell-based therapy, diagnostics, and drug delivery, among others (reviewed in Reference [25]). Exosomes offer a potent mechanism for communication between nearby or distant cells or tissues, to change their physiological functions and properties in particular innate and adaptive immune responses [26]. In mammals, exosomes participate in responses during viral and bacterial infection, in a complex interplay between pathogens and different types of immune cells of the host (macrophages, NK cells, DCs, T and B cells). Exosomes take part in antigen presentation for activation of immune cells and stimulate the release of inflammatory factors and the expression of immune molecules. Exosomes of infected cells can deliver PAMPs (pathogen-associated molecular patterns) and host–derived PRRs (pattern recognition receptors) to bystander cells leading to activation of innate immunity, participating in inflammatory responses and modulation of immune responses [27,28,29,30]. During microbial infection, exosomes can strengthen innate and specific immune responses, and thereby, the immune resistance against the invading microbes, but they can also induce immunosuppression [30].

Exosomes have also been described in bacteria, fungi, and plants [31], as well as in invertebrate model organisms [32]; however, little information is available on their role in immune response in aquatic invertebrates. In the freshwater cnidarian *Hydra vulgaris*, imaging of inward and outward trafficking of functionalised gold nanoparticles, carried out at the whole organism level, identified exosome-like structures that may act as potential carriers to shuttle the nanoparticles in and out the cells, thus participating in the elimination of non-self material from the body [33].

With regards to marine invertebrates, in the Eastern Oyster *Crassostrea virginica*, exosome-like vesicles have been observed in relation to shell formation and repair. When the outer shell of the bivalve is damaged, a thin layer of extracellular matrix is formed by blood cells, the haemocytes, and outer mantle epithelium-OME, into which exosome-like vesicles, which range in diameter from 50 to 500 nm, some containing crystals, are deposited. These structures, identified by epi-fluorescent and laser scanning microscopy, progressively organise into well-defined mineral structures [34]. In another study on deposition of components of the organic shell matrix, membrane bound micron-sized structures stained for chitin were observed, suggesting that exosomal-like structures produced by haemocytes are involved in chitin deposition [35]. Exosomes, also called chitosomes, are well known for the production of chitin in yeast and fungi (see Reference [31]). The role of exosomes in the complex formation of the shell, which is composed of various biomineral ultra-structures and macromolecular organic components, has been further described by Song et al. [36]. The shell matrix proteins, mostly secreted from OME, and partly by haemocytes, are either directly delivered to the mineralisation site via exosome or classical secretory pathway, or first transported to the haemolymph, and then engulfed by granular haemocytes, which will disintegrate and release shell proteins and CaCO_3_ crystals at the mineralisation front. These processes may also be involved in the nucleation and remodelling process of CaCO_3_ mineral.

However, since exosomes also act as innate immune effectors that contribute to host defence [30], recent studies also focused on immune functions of exosomes in marine invertebrates. Marine bivalves and crustaceans possess an open circulatory system, where the body fluid, the haemolymph containing free circulating immune cells, the haemocytes, flows in direct contact with tissues, making it an ideal carrier for exosomes to perform their immune functions during pathogen infection. In *C. gigas*, the transcriptomic analysis was applied to explore the global expression changes of exosomes after stimulation with both Gram (−) and Gram (+) bacteria (*Staphylococcus aureus* and *Vibrio splendidus*, respectively) [37]. Using a RNA-Seq and Ion Torrent Proton System, 1505 abundant exosomal shuttle mRNAs (esmRNAs) were identified. These abundant esmRNAs could be categorised by gene ontology (GO) analysis into 15 cellular components, 12 molecular functions and 21 biological processes, and were mapped onto 62 biological signalling pathways by KEGG. The results showed 68 and 99 significant differentially expressed genes (DEGs) in samples after *S. aureus* and *V. splendidus* challenge, respectively. Identified DEGs, including those related to immune function, showed an extremely high specificity towards different bacterial stimuli. There were four immune-related DEGs potentially involved in responses to *S. aureus* (β-1,3-glucan-binding protein, cathepsin L1, E3 ubiquitin-protein ligase and low-density lipoprotein receptor adapter protein) and 6 in response to challenge with *V. splendidus* (baculoviral IAP repeat-containing protein 2, cathepsin L1, Cu/Zn superoxide dismutase, heat shock 70 kDa protein, high mobility group protein 1 and Toll-like receptor 2). The significant up-regulation of the apoptosis-related gene baculoviral IAP repeat-containing protein 2 indicated that apoptosis played important roles in the oyster’s innate immune response against *V. splendidus* infection mediated by exosomes. These data provide the first information on the role of exosomes in the innate immune response to bacterial infection in a marine invertebrate and on the molecular mechanisms involved [37].

The role of exosomes has also been investigated in relation to viral infection in crustaceans in particular in the Asiatic mud crab (*Scally paramamosain*) upon infection with White spot syndrome virus (WSSV), that causes huge economic losses in aquaculture [38]. MiRNAs from exosomes released from WSSV-injected crabs could suppress viral invasion by inducing apoptosis of haemocytes. Besides, miR-137 and miR-7847 were found to be less packaged in mud crab exosomes during viral infection, with both miRNAs acting as negative apoptosis regulators by targeting the apoptosis-inducing factor (AIF). AIF did not only translocate to the nucleus to induce DNA fragmentation, but could also competitively bind to HSP70 to disintegrate the HSP70-Bax (Bcl-2-associated X protein) complex, which eventually activated the mitochondria apoptosis pathway via free Bax. These findings provide a novel mechanism underlying the crosstalk between exosomal miRNAs and apoptosis pathway in innate immunity in invertebrates.

Given the biological activities of exosomes in intercellular transportation, in communicating information, and in the modulation of cell-mediated immunity after microbial infection, these data underline their role in immune response and apoptotic processes in marine invertebrates and open up further research in comparative and environmental immunology and resistance to disease in key invertebrate species.

Studies carried out in genetic model organism provided the first insight into the in vivo functions of exosomes in invertebrate reproduction, behaviour and development [32]. However, these studies are likely complicated by the diversity of vesicles produced by cells. Although most purification strategies for exosomes focus on extracellular vesicles smaller than 100 nm in diameter, studies in *Caenorabditis elegans* and *Drosophila melanogaster* have shown that the same cells release the same signalling molecules in both exosomes and microvesicles, that is from 30–500 nm in diameter; the same applies to early embryos (see Reference [2] and their references). These data suggest that studies on invertebrate exosomes focusing on the size of vesicles may not reveal their diverse roles in vivo. Thus, it is important to characterise the native repertoire of extracellular vesicles produced by different invertebrate cells types before designing appropriate purification methods to analyse their specific cargo and speculate on the role of exosomes in different physiological functions. These studies may help better understanding the participation of exosomes in invertebrate immunity.

## 4. Tunnelling Nanotubes (TNTs)

Mechanisms of intercellular communication mediated by direct cell contact are important not only among excitable cells (synapses), but also for many immunological processes, such as the formation of the immune synapse between T lymphocyte and antigen-presenting cells that control the physiology of T cell responses [39,40]. However, it is now clear that contact-dependent communication is not always restricted to immediately adjacent cells. Tunnelling nanotubes (TNTs) are thin membranous structures that allow the transfer of signals, from ions to vesicles and organelles [41]. They were first described in cultured rat pheochromocytoma PC12 cells [42] and have subsequently been described in both physiological conditions and response to stress in different mammalian cells types [43] and in almost all immune cells [44,45].

TNTs are long thin F-actin-based membranous channels connecting cells, that can reach lengths over 100 μm, with diameters ranging between 50–200 nm, but also thicker (up to 800 nm), that can contain microtubules. TNTs mediate connections between two cells (of the same or different cell types, i.e., homotypic and heterotypic), but also among several cells, forming networks. Due to the wide range of diameters and lengths of TNTs, to differentiate them from other membrane structures (such as retraction fibres or filopodia), they are identified by three main criteria: (1) They are not attached to the substrate, (2) they attach two cells, and (3) they contain actin [44]. However, the identification of TNTs is limited by the fact that these connections are highly fragile and sensitive to light exposure, shearing force and chemical fixation, and by the lack of known markers. Therefore, TNTs in mammalian cells are generally investigated in live cultures using either a membrane dye, such as FM1-43 or fluorescently labelled wheat germ agglutinin (WGA), or genetically expressing a fluorescent membrane marker [41] and refs therein [46].

Although the processes involved in TNT formation are not completely understood, two main mechanisms have been proposed, both involving actin polymerisation: (1) The actin-driven protrusion mechanism, that involves one or two protrusive events that connect and eventually fuse with the membrane, or the protrusion of the other cell; (2) the cell-dislodgement mechanism of two cells in close contact allowing membranes to fuse [47]. As the cells migrate away from each other, a TNT is formed composed of membrane originating from either one or both cells. The latter mechanism may be typical of motile cells, including immune cells (e.g., macrophages or lymphocytes). A number of proteins involved in actin polymerisation, as well as proteins involved in immune signalling, have been identified in TNTs [48,49]. TNT formation also requires a distortion of the plasma membrane, and several model mechanisms have been described for the rearrangement of lipids that can explain the different morphologies observed, according to the combination of forces applied and components, such as the curvature changes and cytoskeletal forces that contribute to membrane deformation [50].

Moreover, some uncertainties apply to the processes involved stimulation or induction of TNT formation in different cells types, including immune cells. Macrophages and dendritic cells show a basal level of TNT formation. Stimulation has been observed with pro-inflammatory signals either applied exogenously or during pathogen infection [51,52]: However, there do not seem to be dramatic differences between different stimuli. For example, exposure to IL-2, IL-12, IL-15, and IL-18 increased the number of TNTs in NK cells; however, a similar increase was observed with different stimulating cytokines [52]. However, the “basal level” of nanotube formation in different immune cells is unknown, since the presence of different components in normal serum/media utilised for mammalian cell culture (including growth factors) may hamper the effects of added stimulants.

In the last decade, TNTs have been shown to have different functional roles in physiological and pathological processes, such as signal transduction, micro and nano-particle delivery, immune responses, embryogenesis, cellular reprogramming, apoptosis, cancer, initiation and progression of neurodegenerative diseases, and pathogens transfer (reviewed in Reference [43]). All include the transfer of cargo or signals from one cell to another: TNTs have been shown to transfer mitochondria or vesicles derived from early endosomes, endoplasmic reticulum, Golgi complex, and lysosomes; plasma membrane and membrane-associated proteins; cytoplasmic proteins and signalling molecules, including [Ca]^2+^ signals. However, the biological significance of TNTs in the function of different types of immune cells is far from being elucidated. The majority of the established biologically significant contributions of TNTs involve transmission and infection of foreign agents, such as prion and HIV [43]. Further studies are needed to clarify the physiological role of TNTs in normal immune surveillance, maintenance, or activation of immune functions.

TNTs have been investigated so far mainly in mammalian cells. Although described in models from other vertebrate taxa (avian, amphibians, fish), TNT-like structures have been generally observed during embryonic development; however, their study also appears complicated by the need to distinguish them from other similar structures like cytonemes or intracellular bridges (reviewed in Reference [53]). Moreover, structures analogous to TNTs and with roles in intercellular communication can be found across phylogenetic taxa, such as plasmodesmata that connect two adjacent cells in plants, or septa in fungi [54]. In invertebrates, the presence of these structures has been solely reported in *Drosophila* cell lines [55], where TNT-like structures were identified, positive for F-actin and tubulin staining, non-adherent, and containing components of the RNA interference (RNAi) system, including Ago2, dsRNA, Rab7, and CG457239. The role of TNTs was demonstrated during the antiviral response, where infected cell showed a significant increase in TNT number, allowing the spread of antiviral signals (e.g., Argonaute 2 RNA interference-RNAi) from infected to non-infected cells, to achieve an effective immune response. This suggests the participation of nanotube-like structures in establishing systemic RNAi antiviral immunity in *Drosophila* [55].

The existence of such comparable structures in *Drosophila* increases the probability to encounter TNTs-like structures involved in cell communication in other invertebrate species. In particular, TNTs may play a role in invertebrate cells responsible for innate immunity, that share many features with those of vertebrates. However, the presence of TNT in invertebrate immunocytes has not been described yet.

In marine bivalves, haemocytes have been thoroughly characterised in terms of functional responses and immune gene expression [56]. Despite the number of data obtained by different microscopical techniques, devoted to identifying haemocyte subpopulations, intra and extracellular structures and molecules, the formation of TNT-like structures in bivalve haemocytes has been neglected so far. In this light, attention was turned on the possible presence of TNTs in the haemocytes of the marine mussel *Mytilus galloprovincialis*, that have been widely utilised as an in vitro model for cell-mediated immunity in invertebrates [57]. Mussel haemocytes, due to their role in innate immunity and wound healing, have a strong tendency to adhere to different substrates and to aggregate, forming cell clumps; moreover, they normally show several intercellular connections when observed by simple optical microscopy, including short filopodia (Supplementary Material Figure S1). Moreover, haemocytes are highly motile cells [58], with a mean velocity of 2.78 μm/min, comparable with that of human neutrophils, and that can reach up to 7 μm/min, which increases the possibility of establishing multiple contacts in both physiological and stress conditions.

We first looked for the possible presence of TNT-like structures in *Mytilus* haemocytes by simple light microscopy in both fixed cells stained with Giemsa and in live cells loaded with the vital dye Neural red (NR). In all experiments, filter sterilised artificial seawater (ASW) was utilised as an external medium. *Mytilus* haemocytes are mostly represented by granular cells, containing several acidic vacuoles, which can be easily visualised by NR staining; accordingly, the NR retention time assay is widely utilised as a marker of cell health [59]. As shown in Figure S1, a number of intercellular connections of variable length and width can be observed, both between two haemocytes or between individual haemocytes and cell clumps.

**Figure 2:**
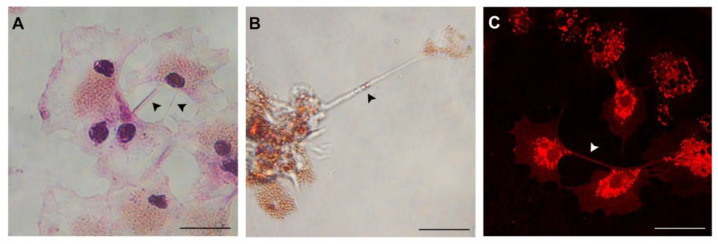
TNT-like structures between the haemocytes of *M. galloprovincialis* observed with different stainings. (**A**) fixed haemocytes stained with Giemsa; live haemocytes stained with Neutral Red (NR) for lysosomes (**B**), and with the Fluorescent dye Tetramethylrhodamine ethyl ester perchlorate (TMRE), fro mitochondria (**C**). See Supplementary Materials for details. Scale bar: 20 µm.

As shown in detail in Figure 2, some thin and transparent connections between haemocytes, about 20 µm in length, can also be observed in cells stained with Giemsa kit after a mild fixation in methanol (Figure 2A, arrowheads). More details on these intercellular connections can be obtained with different dyes. In Figure 2B a representative image of live, NR loaded haemocytes is displayed: A long TNT-like tube (>40 µm) connecting an isolated haemocyte with a cell clump can be observed; this structure is on a different plane of focus from that of adherent haemocytes and contains small NR loaded vesicles, representing lysosomes (Figure 2B, arrowhead). In live haemocytes stained with TMRE (Tetramethylrhodamine ethyl ester perchlorate), a marker for the mitochondrial membrane potential, some mitochondria can be seen at the emergence of the connection between two cells, suggesting that also these organelles may be exchanged (Figure 2C).

When observed by live imaging, the formation of these TNT-like structures occurs spontaneously. The process more commonly observed so far in NR loaded haemocytes involves the progressive distancing of individual haemocytes from small clumps or of two cells, as well as the transfer of cargo in the form of lysosomal vesicles, the whole process taking about 6 min (see Supplementary Video S1). Whatever the type of connection formed, TNT-like structures generally emerge from the perinuclear area of the haemocyte, and on the side of the cell membrane devoid of other structures, such as short filopodia (Figure 2 and Video S1). Although these processes occur spontaneously, it must be taken into account that they may be affected by experimental stress factors during microscopic observations, particularly light.

Overall, these observations suggest that TNT-like structures may represent an important route for intercellular communication among mussel haemocytes. In this light, research is needed to investigate their role in immunity and stress response. Interestingly, these observations are made in live haemocytes freshly extracted from healthy adult mussels, in the presence of artificial seawater-ASW as an extracellular medium. This offers the possibility to investigate possible TNT induction/stimulation or inhibition by virtually any kind of stimuli. The main limitation in the use of bivalve haemocytes, that is the absence of established protocols or suitable culture media [57] can thus turn out in an advantage for investigating TNT formation in different experimental conditions.

Although over the last decade, research has effectively improved our understanding of TNTs and their role in cell-to-cell communication, their structural identity and complexity remain largely unknown. The main open questions are (1) whether these protrusions are different from other cellular processes, such as filopodia and (2) whether their function in the exchange of cargos between cells is due to direct communication between the cytoplasm of distant cells or to a classic exo/endocytosis process (see Reference [60] and their references). Recent studies utilising a combination of live imaging, correlative light- and cryo-electron tomography approaches to study neuronal TNTs, revealed their distinct structural features. In particular, it has been shown that neuronal TNTs are composed of a bundle of open-ended individual nanotubes (iTNTs) that are held together by threads labelled with anti-N-cadherin antibodies. iTNTs are filled with parallel actin bundles on which different membrane-bound compartments and mitochondria appear to transfer. In addition, by using correlative focused-ion beam SEM (FIB-SEM) it was shown that TNTs can be open on both ends [60]. Overall, this study showed that TNTs are a distinct cellular structure when compared to other membrane protrusions and identified their structural features, supporting their role in allowing direct communication between the cytosol of distant cells and cargo transfer. Further work is needed to unravel the complexity of TNTs formed by immune cells. In this light, invertebrate models of innate immunity may provide a tool for investigating TNT structure and function.

## 5. Conclusions

From this short review of available literature, evidence is provided for the presence of different mechanisms of intercellular communication that are shared among vertebrate and invertebrate cells and that are involved in several key biological processes, from immune defence to development.

In invertebrates, where the immune response is limited to innate immunity, the capacity to coordinate and orchestrate the immune response among cells through different mechanisms can offer multiple possibilities of defence against non-self. This further complexity and plasticity of the immune response could contribute to the ability of invertebrates to survive in complex natural environments, only relying on the cell-mediated immune response. Available data supporting the presence of ETosis, exosomes, and TNTs in aquatic invertebrates are summarised in Table 1. In particular, in the immunocytes of marine species living in coastal areas, that are exposed to multiple environmental fluctuations, these intercellular communication systems may offer a further advantage in the defence against potential pathogens, and in general, to non-self-particles. In this light, different types of communication systems may even play major roles in invertebrate immunity than those so far identified in mammalian systems.

However, experience from mammalian models has taught us how these structures can be extremely fragile, or transient, or produced in minute amounts: Therefore, the possibility to identify them, their components and functional roles in marine invertebrate cells, is still limited by the technical constraints that have made their identification and characterisation not straightforward also in mammalian cells. On the other hand, the utilisation of immunocytes from well-known invertebrate models, such as bivalve molluscs, may represent a valuable tool for in vitro studies on different intercellular communication systems under a variety of physiological and stress conditions. Therefore, further research is needed to develop suitable experimental protocols adapted to marine invertebrate immunocytes to clearly identify extracellular NETs, exosomes and TNTs. A nice example of this is the work carried out on ETosis in mussel immunocytes [19]. These protocols may also help to reveal the physiological role of these intercellular communication systems in vivo under a variety of stimuli, from challenge with different pathogens or foreign particles, to exposure to chemicals and changes in environmental variables. Despite the evidence provided so far on the existence of these three communication methods in invertebrates, further research is needed to define their role and functions in immunity.

Immune cells must communicate to perform physiological functions also in the absence of infection: In the course of infection, they must interact with each other in order to activate a proper immune response [41]. In comparative immunology, research on invertebrates has long been focused on the search for the main soluble means of communication in the long- and short-range (cytokines, chemiokines, growth factors), in analogy with vertebrate systems. However, studies on invertebrate ETs, exosomes, and TNTs as other methods of communication, in addition to soluble factors, represent a challenging field of research. Although each of this method has unique properties, in mammalian systems interactions have been observed between exosomes and TNTs [41,61], as well as exosomes and NETs [62]. Mast cells have been shown to interact with the nervous system through exosomes, TNTs and ETs, with implications across a variety of pathological conditions [63]. Elucidating the mechanisms of these novel and alternative means of communication and their possible interactions in invertebrates will provide critical new insights into invertebrate immunity in both physiological and pathological conditions.

## Figures and Tables

**Table 1:** Summary of available information on different intercellular communication systems in aquatic invertebrates.

	Species	Cell Type	Stimulus	Observations	Reference
ETosis	Crab (*Carcinus maenas*)	Haemocytes	PMA, LPS, *Listonella anguillarum*	DNA release	[16]
	Mussel (*Mytilus edulis*)	Haemocytes	PMA	DNA release	[16]
	Sea anemone (*Actinia equina*)	Mesogleal cells	PMA	DNA release	[16]
	Oyster (*Crassostrea gigas*)	Gill and muscle cells, Haemocytes	In vivo: *Vibrio tasmaniensis* In vitro: *V. tasmaniensis, zymosan*, PMA	Release of DNA networks, antimicrobial H1-H5-like histones, ROS, bacterial entrapment	[17]
	Pacific White Shrimp (*Litopenaeus vannamei*)	Haemocytes	PMA, LPS *E. coli*	DNA release, bacterial entrapment, antimicrobial proteins	[13]
	Pacific White Shrimp (*Litopenaeus vannamei*)	Haemocytes	PMA, LPS *E. coli*	Release of DNA, antimicrobial proteins, role of ETs and phagocytosis depending on bacterial density	[14]
	Kuruma shrimp (*Marsupenaeus japonicus*)	Haemocytes	PMA, LPS, PGN, *E. coli*	Chromatin release, lysozyme release, entrapped *E. coli*	[15]
	Ascidian (*Botryllus schlosseri*)	Cytotoxic morula cells, phagocytes	yeast, zymosan, LPS, *Bacillus clausii*	Release of amyloid fibrils	[19]
	Mussel (*Mytilus* *galloprovincialis*)	Haemocytes	zymosan, glucans, PG, LTA, polyI:C, PMA, flagellin, *V. splendidus*, UV light, A23187	DNA release, total and mitochondrial ROS production	[20]
Exosomes	Cnidarian (*Hydra vulgaris*)	Ectodermal cells	gold nanoparticles (AuNPs)	Exosomes containing AuNPs for elimination of nonself material	[33]
	Oyster (*Crassostrea virginica*)	Haemocytes/ epithelial cells	Shell injury	Exosome-like vesicles containing calcite crystals, deposited to the extracellular matrix	[34]
	Oyster (*Crassostrea virginica*)	Mantle epithelial cells/ haemocytes	Shell injury	Transport and chitin deposition	[35]
	Oyster (*Crassostrea gigas*)	Haemocytes	In vivo challenge with *Staphylococcus aureus* and *V. splendidus*	Exosomal shuttle mRNAs (esmRNAs) involved in immune function	[37]
	Mud crab (*Scylla paramamosain)*	Haemocytes	In vivo challenge with white spot syndrome virus WSSV	miRNA (miR-137, miR-7847) suppress viral invasion inducing apoptosis of haemocytes	[38]
TNTs	Mussel (*Mytilus* *galloprovincialis*)	Haemocytes	none	formation in live cells, recording, exchange of lysosomal vesicles, mitochondria?	[this work]

## Supplementary Materials

The following are available online at https://www.mdpi.com/2079-7737/9/8/234/s1, Figure S1: General overview of freshly isolated haemocytes from *Mytilus galloprovincialis*, showing both individual cells and small cell clumps. Video S1: TNT-like structures in live haemocytes from *Mytilus galloprovincialis.*
