# Supplementary material for: Conservation of Cell Communication Systems in Invertebrate Host–Defence Mechanisms: Possible Role in Immunity and Disease
Source: Biology (Basel). 2020 Aug 18;9(8):234. doi: 10.3390/biology9080234 (PMC7464772; doi:10.3390/biology9080234)
Supplement: Supplementary file 1 [file biology-09-00234-s001.pdf]

Supplementary Material

Conservation of cell communication systems in invertebrate host–defence mechanisms: possible role in immunity and disease

Manon Auguste [1], Teresa Balbi [1], Caterina Ciacci [2] and Laura Canesi [1,*]

[1] Department of Earth Environment and Life Sciences (DISTAV), University of Genoa, Genoa, Italy; manon.auguste@edu.unige.it (M.A.); teresa.balbi@unige.it (T.B.)

[2] Department of Biomolecular Sciences (DIBS), University 'Carlo Bo' of Urbino, Urbino, Italy; caterina.ciacci@uniurb.it (C.C.)

* Correspondence: laura.canesi@unige.it (L.C.)

Methods

Mussels and hemocyte monolayers

Mussels (Mytilus galloprovincialis Lamarck, 1819), 4–5 cm long, were purchased from a local aquaculture farm (La Spezia , Italy) in May 2020 and kept for 1 day in static tanks containing aerated artificial sea water (ASW), salinity 36 ppt (1 L/mussel) at 18 °C. Hemolymph was extracted from the posterior adductor muscle using a sterile 1 mL syringe with an 18 G1/2″ needle. With the needle removed, hemolymph was filtered through a sterile gauze and pooled in Falcon tubes at 18 °C. Hemocyte monolayers were prepared using 20 µL whole hemolymph dropped on a glass slide and the cells were let to adhere for 20 min to the support in a humid chamber at 18 °C (Ciacci et al., 2012). After adhesion, hemocytes were washed out with 20 µl ASW and visualized with different staining.

Giemsa staining: the Giemsa kit (Diff-QuikTM, Medion Grifols Diagnostics AG, Switzerland), was used following the manufacturer's instructions. Monolayers were first fixed using the kit fixative solution (>50% methanol), and then stained for the dye mixture (Stain Solution I containing Eosin Y; Stain Solution II containing Thiazine Dye) and observed under light microscope.

Lysosomal staining: hemocytes were incubated with 20 µL of a neutral red (NR) (Sigma-Aldrich, Milan, Italy) solution (final concentration 40 µg/mL in ASW from a stock solution of NR 20 mg/mL DMSO - dimethylsulfoxide). After 15 min, excess of dye was washed out, samples were added with 20 µL filtered ASW and observed.

Mitochondrial staining: hemocytes were incubated with the fluorescent dye Tetramethylrhodamine ethyl ester perchlorate (TMRE) (40 nM in ASW for 10 min) as previously described (Ciacci et al., 2012) and then observed by confocal microscopy (Exc = 488 nm; Em = 580 nm).

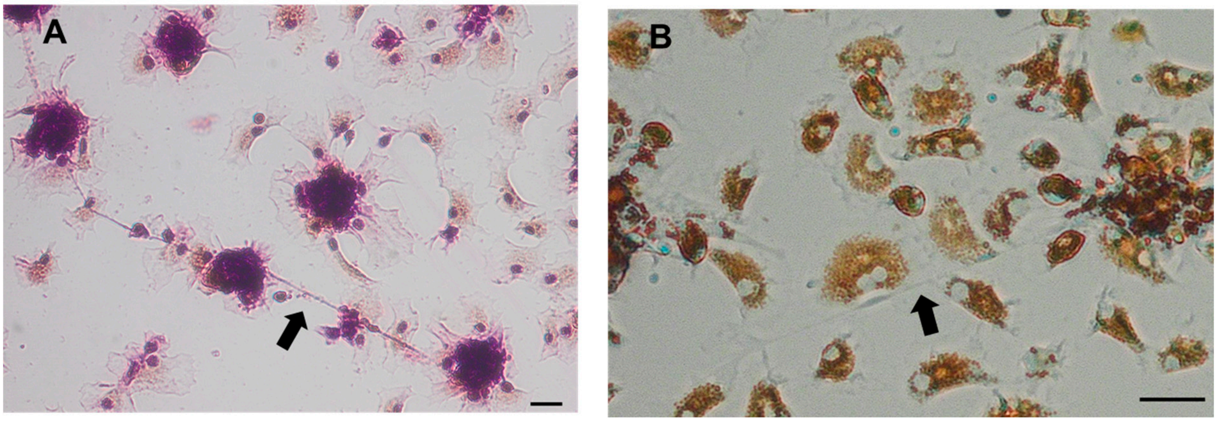

Figure S1. General overview of freshly isolated hemocytes from *Mytilus galloprovincialis*, showing both individual cells and small cell clumps. A) Fixed hemocytes stained with Giemsa.; B) Live hemocytes loaded with Neutral Red (NR), staining lysosomes. In both images, different types of connections among individual cells and between isolated cells and cell clumps can be observed in particular, long connexions resembling TNTs-like structures between cells and small clumps can be observed (arrow). Scale bar: 20μm.

Reference

1. Ciacci, C., Canonico, B., Bilanicova, D., Fabbri, R., Cortese, K., Gallo, G., Marcomini, A., Pojana, G., Canesi, L., 2012. Immunomodulation by different types of n-oxides in the hemocytes of the marine bivalve Mytilus galloprovincialis. PLoS One 7, e36937.

Legend to Supplementary Video S1

TNT-like structures (white arrow) in live hemocytes from Mytilus galloprovincialis. At least 3 thin transparent connections can be observed between hemocytes that are out of the plane of focus with respect to cells; in the one in the middle, the exchange of a cargo containing vesicles lysosomes (stained with NR) between cells can be observed. Video 4x speed. Scale bar: 200 pixels= 20 μm.
